# Was It a Consideration for the Welfare of the Inmates,—or Politics?

**Published:** 1890-09

**Authors:** 


### WAS IT A CONSIDERATION FOR THE WELFARE OF THE INMATES,—OR POLITICS?

The position of first assistant physician at the State Lunatic Asylum, at Austin, made vacant by the resignation of Dr. John Preston, mentioned elsewhere in this issue of the JOURNAL, has been filled by the appointment, by Superintendent Dorset, of Dr. R. R. Walker, of Paris, Texas,—the Board of Directors confirming the appointment. Dr. Walker, we learn, is a young man of thirty, and comes well recommended; no doubt he will make a worthy successor to Dr. Preston and fill the position satisfactorily; but under existing circumstances it would seem pertinent to enquire why Dr. J. A. Davis, the present second assistant physician, was not promoted to thé vacancy? Leaving out of consideration any other claims Dr. Davis may have had to preferment, he possesses the advantage of having had several years experience in the treatment of the insane, while Dr. Walker, we understand, like his ranking officer, Supt. Dorset at the time of his appointment, is totally inexperienced with the insane. It has always been a mystery to the JOURNAL, and to others of the uninitiated, how, in the confirmation of Dr. Dorset, the Board, and in his appointment, the Governor *got over* or dodged around that clause of the law which distinctly stipulates that the person selected for Superintendent of the Lunatic Asylum *shall have had experience* in that line of practice. Dr. Dorset, we have always understood from reliable authority, never saw the inside of an asylum till Governor Ross chucked him into Dr. Denton's place for no other reason, so far as we are imformed, than that "he came well recommended,"—(not by the medical profession, however)—and after the board had committed themselves to Dr. Denton for, and he had actually entered upon a second term.

We do not know whether the law requires experience at the hands of the first assistant physician,—and therefore, whether or not it has *again* been ignored in this appointment; but, whether required by law or not, it would seem that if the welfare of the patients be the object of the law regulating said appointments, preference should be given to experienced physicians in all cases; *cæteris paribus*.

That personal ill feeling exists between Drs. Dorset and Davis is well known hereabout; but, personal dislike on the part of a superintendent toward a subordinate officer should not blind him to the rights of the latter to promotion, if he be meritorious and qualified; and there must be some reason why Dr. Davis was passed over, and a new man, and we learn an inexperinced man, assigned to the position, thus becoming his ranking officer.

This inquiry becomes the more pertinent and emphatic from the circumstance that two years ago both these gentlemen—Davis and Walker—were applicants for the second physician's position. Dr. Walker was nominated by the superintendent as his choice, and the Board rejected him, or refused to appoint him, which is the same thing, whereupon Dr. Dorset sent in the name of Dr. Davis, and he was confirmed, and has filled the position to date. Why this same Board should now accept Dr. Walker for the *first* position, instead of Davis, will, we suppose, remain one of those mysteries "which no fellow can find out." But such things are liable to occur where offices of the kind are subject to political influences; and this striking case but emphasizes the necessity for a law which shall remove the eleemosynary institutions of the State from the domain of politics, as suggested by the JOURNAL in a former issue. It is certainly very discouraging to a junior officer, to be made thus emphatically to realize that he stands no chance for promotion, if the "Boss" doesn't like him, —or that he hasn't a 'pull' on the right political string.

